# An exploratory study of problematic shopping and problematic video gaming in adolescents

**DOI:** 10.1371/journal.pone.0272228

**Published:** 2022-08-10

**Authors:** Norman R. Greenberg, Zu Wei Zhai, Rani A. Hoff, Suchitra Krishnan-Sarin, Marc N. Potenza

**Affiliations:** 1 Yale School of Medicine, New Haven, Connecticut, United States of America; 2 Program in Neuroscience, Middlebury College, Middlebury, Vermont, United States of America; 3 Department of Psychiatry, Yale School of Medicine, New Haven, Connecticut, United States of America; 4 Connecticut Mental Health Center, New Haven, Connecticut, United States of America; 5 Connecticut Council on Problem Gambling, Wethersfield, Connecticut, United States of America; 6 Child Study Center, Yale School of Medicine, New Haven, Connecticut, United States of America; 7 Department of Neuroscience, Yale University, New Haven, Connecticut, United States of America; Mayo Clinic College of Medicine, UNITED STATES

## Abstract

Problematic video gaming (PVG) and problematic shopping (PS) are addictive behaviors prevalent in adolescents, characterized by positive and negative reinforcement, and associated with psychosocial impairment. This study examined how PS and PVG relate in adolescents. It also examined how PS interacts with PVG in relation to health/functioning measures. Survey data from 3,657 Connecticut high-school students were evaluated. Chi-square analyses and logistic regression models were used to assess relationships between PS and measures of PVG. Interaction analyses measured effects of PS on relationships between PVG and health/functioning measures. Relative to adolescents without PS, those with PS had 8.79-fold higher odds of exhibiting PVG and were more likely to endorse gaming to relieve anxiety and impairment due to gaming. Interaction analyses revealed that in adolescents with PS, the relationships between PVG and aggressive behaviors, including fighting, serious fighting leading to physical injury, and weapon-carrying, were stronger than in adolescents without PS. PS strongly relates to PVG, and among youth reporting PS, there are stronger associations between PVG and aggressive behaviors. Prevention efforts for adolescents should consider the co-occurrence of PS and PVG. PS and PVG may be linked by negative reinforcement and propensities for aggressive and addictive behaviors, suggesting that further research should explore possible interventions targeting stress management and maladaptive coping.

## Introduction

While video gaming is a common recreational activity among adolescents, problematic patterns of video gaming have been recognized as a growing public health concern [1]. Problematic video gaming (PVG) refers to excessive and poorly controlled engagement in video gaming leading to psychosocial and functional impairment [2]. PVG has been associated with substance use, social anxiety, depressed mood and dysphoria, poor school performance, and violent behaviors [3–7]. Due to impairment and distress related to PVG, internet gaming disorder was included in the fifth edition of the Diagnostic and Statistical Manual (DSM-5) as a condition for further study [8], and gaming disorder was included in the eleventh revision of the International Classification of Diseases (ICD-11) [9].

PVG has been considered an addictive behavior given features that include preoccupation with gaming, poor behavioral control over gaming, strong urges for gaming, unsuccessful attempts to cut back, rising tension/anxiety just prior to gaming, and continued gaming despite associated impairment [3, 6, 10]. Based on epidemiologic and imaging studies, the Interaction of Person-Affect-Cognition-Execution (I-PACE) model has been proposed to describe the development of behavioral addictions, including PVG [11, 12]. The I-PACE model posits that addictive behaviors result from the interaction of predisposing characteristics (such as sensation-seeking) in individuals, their environments, affective or cognitive responses to stimuli (such as negative mood), and poor inhibitory control. The I-PACE model further suggests that PVG may progress from motivations involving positive reinforcement, such as seeking excitement or joy, to motivations involving negative reinforcement, or alleviation of anxiety and tension, in later stages [11]. This is consistent with previous studies that have shown mood modification and negative reinforcement as being relevant to PVG [2, 13].

Similar to PVG, problematic shopping (PS) refers to poor control over shopping behavior, with repeated shopping episodes despite negative impairment, such as large debts [14]. PS is characterized by many of the core features of behavioral addictions, including preoccupation about shopping, poor control over urges to shop, exhilaration during shopping episodes, relief of rising tension or anxiety by shopping, and impairment due to shopping [14–17]. Like PVG, negative reinforcement, or shopping to relieve anxiety or tension, contributes to PS [2, 18]. In addition to sharing features with other behavioral addictions, PS has been associated with addictive and impulse control disorders (ICDs) and related behaviors, such as substance-use disorders, kleptomania, gambling disorder, and repetitive self-injurious behaviors [16–19]. Like PVG, PS has also been associated with dysphoria, interpersonal conflict, and violent behaviors, such as weapon-carrying and physical fighting [17, 20]. Additionally, PS and aggression have both been associated with borderline personality disorder and a history of childhood trauma, further supporting relationships between the behaviors [21–23].

Though PS and PVG share many similar features including positive and negative reinforcement motivations, difficulties with impulse control, associations with substance and behavioral addictions and aggressive behaviors, their relationships to one another and related clinically relevant domains are not well understood, especially in adolescents. An association between problematic use of the internet and PS has previously been shown, although a specific association between PS and PVG was not explored [24]. Problem behavior theory, articulated by Jessor, posits that problematic behaviors in adolescents often occur as part of a syndrome of behaviors with associated negative effects on health and functioning, rather than in isolation [25]. Further, poor impulse control often underlies PVG and PS, possibly driving co-occurrence of the behaviors [13, 14]. Understanding whether PS and PVG associate is important for prevention efforts of each entity, as adolescents with one condition may be more thoroughly screened for the other. Additionally, in line with problem behavior theory, the co-occurrence of PVG and PS may be associated with more negative health consequences and other problem behaviors (like violent behaviors or substance use). Therefore, it is important to identify relationships between PS, PVG and measures of health and functioning.

To address these gaps in the literature, we analyzed survey data from 3,657 high school students in Connecticut. Survey data contained information about the adolescents’ engagement in PS, PVG, aggressive behaviors, substance use, and other behaviors. Due to the shared features of behavioral addictions present in PS and PVG, we posited that PS and PVG would be positively associated among adolescents. We also hypothesized that PS would associate with individual components of PVG including urges for video gaming, attempts to reduce video gaming, gaming to relieve anxiety, self-reported problems with gaming, and missing activities for video gaming. Next, exploratory analyses were performed to assess whether co-occurring PS influenced relationships between PVG and associated heath/functioning correlates, such as substance use and aggressive behaviors. Lastly, due to the role of negative reinforcement in addictive behaviors, particularly for females and racial/ethnic minority groups [2, 26], we performed exploratory analyses to investigate relationships between shopping to relieve anxiety or tension (STRAT) and measures of PVG, as well as gaming to relieve anxiety or tension (GTRAT) and measures of PS.

## Materials and methods

### Participants

Cross-sectional survey data collected in 2006 from public high-school students in Connecticut were analyzed. As described in previous reports, all public high schools in Connecticut were invited to participate [3]. Parents were notified by mail of the survey and how to exclude their child’s/children’s participation, as detailed below in the ethics statement. Research staff was present on site to notify students that participation was fully voluntary and to distribute, explain, and collect surveys.

Survey data were collected from 4,523 students with a 154-item questionnaire about student demographics and participation in several risky behaviors. Of the students surveyed, 3,657 students (1,643 boys and 2,014 girls) were included in the study. Students were excluded for omitting questions about sociodemographic information (n = 184), shopping behavior (n = 522), and video-gaming behavior (n = 160). The dataset used for the analyses can be found in S1 Dataset.

### Measures

#### Sociodemographic information

Sociodemographic information assessed in the survey included gender, grade-level (9^th^- 12^th^ grade), race/ethnicity (including Black/African American, White/Caucasian, Asian, Hispanic, or “Other”), and family structure (including two-parent household, single-parent household, and “other” family structure).

#### PS measures

PS was assessed as previously described [18]. Participants were asked six “yes” or “no” questions: (a) *Have you ever tried to cut back on shopping?*, *(b) Has a family member ever expressed concern about the amount of time you shop or the amount of money you spend shopping?*, (c) *Have you ever missed school*, *work*, *or other important social activities because you were shopping?*, (d) *Do you think you have a problem with excessive shopping?*, (e) *Have you ever experienced an irresistible urge or uncontrollable need to shop?*, and (f) *Have you ever experienced a growing tension or anxiety that can only be relieved by shopping?*. Questions were derived from the Minnesota Impulsive Disorders Inventory (MIDI), a valid and reliable instrument for assessing PS in adolescents and adults [20, 27, 28]. As previously described, participants who endorsed all three of questions (a), (e), and (f) were categorized as having PS, and those who did not endorse all three questions were classified as not [18]. Participants who endorsed question (f) were included in the STRAT group, and those who did not were included in the non-STRAT group.

#### PVG measures

PVG was determined as previously [3]. Participants were asked six “yes” or “no” questions: (a) *Have you ever tried to cut back on playing video or computer games?*, (b) *Has a family member ever expressed concern about the amount of time you play video or computer games?*, (c) *Have you ever missed school*, *work*, *or other important social activities because you were playing video or computer games?*, (d) *Do you think you have a problem with excessive video or computer game use?*, (e) *Have you ever experienced an irresistible urge or uncontrollable need to play video or computer games?*, and (f) *Have you ever experienced a growing tension or anxiety that can only be relieved by playing video games?*. As previously described, adolescents who endorsed all three questions (a), (e), and (f) were included in the PVG group, while those who did not endorse all three were included in the non-PVG group [3]. Participants who endorsed question (f) were included in the GTRAT group, and those who did not were included in the non-GTRAT group.

#### Health and functioning

Health and functioning were assessed as previously [29]. Engagement in extracurricular activities was assessed by asking about participation in church activities, community service, school clubs and team sports. Participants who endorsed any of the extracurricular activities were compared to those that did not endorse any extracurricular activities. Presence of lifetime marijuana use, drug use, alcohol bingeing, problem with alcohol, and regular smoking were assessed by self-report. Additionally, dysphoria/depression was assessed by asking: *During the past 12 months*, *did you ever feel so sad or hopeless almost every day for two weeks or more in a row that you stopped doing some usual activities?*. Additionally, aggressive behaviors were assessed, including carrying a weapon in the past month, having a physical fight in the past year, or having a serious fight requiring medical treatment in the past year. Although validated screening instruments were not used for the assessment of all health and functioning measures, efforts were made to use items employed in other youth surveys (e.g., the Youth Risk Behavior Survey) to promote comparability across studies.

### Statistical analyses

Reliability analysis of PVG and PS measures was performed by calculating the Kuder-Richardson coefficient using PSPP (https://www.gnu.org/software/pspp/). Statistical analyses were performed using the programming language R. The script used for the analyses can be found in S1 Text. Chi-squared analyses with Yates’ continuity correction were performed to assess sociodemographic differences between PS and non-PS groups, STRAT and non-STRAT groups, GTRAT and non-GTRAT groups, and PVG and non-PVG groups. For analyses of groups with participant counts less than five, Fisher’s exact test was used in place of chi-squared analyses. Logistic regressions were performed to determine the relationships between PS status and video gaming measures. Logistic regressions were also performed to determine the relationships between each shopping measure and PVG status. Further, exploratory logistic regressions were performed to investigate the relationships between STRAT and each PVG measure, as well as GTRAT and each PS measure. Lastly, exploratory binomial logistic regressions were performed to determine whether relationships between PVG and health and functioning, including substance use, extracurricular involvement, dysphoria, and aggressive behaviors differed between those with and without PS. All regression models adjusted for gender, race/ethnicity, grade level, and family structure. Odds ratios (ORs) with 95% confidence intervals (95%CIs) and p-values were calculated for each model. Models with 95%CIs that did not overlap with 1 and p-values < 0.05 were considered statistically significant.

### Ethics

The high-school survey and procedures were approved by the Yale School of Medicine IRB, and all procedures were approved by the participating high-schools. Passive consent procedures were adopted for parental consent. Parents of students were notified by mail of the survey and that to exclude their child’s participation in the study, they should contact the school or the study team. Parental permission for their child’s participation was implied if they did not make contact with the team or school. Students were informed at the time of survey administration that it was being used for a study, that their participation was fully voluntary, and that they could refuse to fill out the survey if they wished. Those who did not participate in the survey were allowed to do school work while others worked on the survey. Students were also told not to include identifying information on the survey to maintain anonymity. Students were given a pen to fill out the survey. Procedures were in accordance with the Declaration of Helsinki (2013).

## Results

### Internal reliability analysis

The three measures used to determine PS had moderate reliability with a Kuder-Richardson coefficient of 0.59. The three measures used to determine PVG had similar, numerically better reliability with a Kuder-Richardson coefficient of 0.63.

### Sociodemographic characteristics

PS was present in 2.4% of the sample (n = 88). The final sample studied included the following racial/ethnic breakdown: 2770 (75.74%) White/Caucasian participants, 255 (6.97%) Black/African American participants, 126 (3.45%) Asian participants, 300 (8.20%) Hispanic participants, and 206 (5.63%) Other (including Native American, Pacific Islander, and Middle Eastern) participants. Table 1 displays the sociodemographic characteristics of adolescents with and without PS. Adolescents with versus those without PS were more likely to be female, be in higher grades, and have experienced PVG.

**Table 1:** Sociodemographic characteristics of adolescents with and without problematic shopping.

	Non-Problematic Shopping (N = 3569)		Problematic Shopping (N = 88)			
Dependent Variable	N	%	N	%	χ2	p-value*
Gender					17.05	<0.001
Male	1623	45.47%	20	22.73%		
Female	1946	54.53%	68	77.27%		
Race/Ethnicity						
White/Caucasian					0.63	0.43
No	862	24.15%	25	28.41%		
Yes	2707	75.85%	63	71.59%		
Black/African-American					0.02	0.88
No	3321	93.05%	81	92.05%		
Yes	248	6.95%	7	7.95%		
Asian					0.08	0.78
No	3446	96.55%	85	96.59%		
Yes	123	3.45%	3	3.41%		
Hispanic					0.8	0.37
No	3279	91.87%	78	88.64%		
Yes	290	8.13%	10	11.36%		
Other					<0.001	1.00
No	3368	94.37%	83	94.32%		
Yes	201	5.63%	5	5.68%		
Grade					8.02	0.046
9th	1093	30.62%	19	21.59%		
10th	976	27.35%	19	21.59%		
11th	944	26.45%	30	34.09%		
12th	556	15.58%	20	22.73%		
Family Structure					3.73	0.15
One parent	818	22.92%	21	23.86%		
Two parents	2582	72.35%	59	67.05%		
Other	169	4.74%	8	9.09%		
Problematic Video Gaming					11.65	<0.001
Non-Problematic Video Gaming	3497	97.98%	81	92.05%		
Problematic Video Gaming	72	2.02%	7	7.95%		

* p-values were calculated using chi-squared tests with Yates’ continuity correction

PVG was present in 2.2% of the sample (n = 79). Sociodemographic characteristics of adolescents with and without PVG are displayed in Table 2. Adolescents with versus those without PVG were more likely to have experienced PS, be male, and be of Asian descent. Among adolescents with PVG, those with concurrent PS were more likely to report being female than those without PS, as shown in Table 3.

**Table 2:** Sociodemographic characteristics of adolescents with and without problematic video gaming.

	Non-PVG (N = 3578)		PVG (N = 79)			
Dependent Variable	N	%	N	%	χ2	p-value*
Gender					44	<0.001
Male	1578	44.10%	65	82.28%		
Female	2000	55.90%	14	17.72%		
Race/Ethnicity						
White/Caucasian					3.79	0.051
No	860	24.04%	27	34.18%		
Yes	2718	75.96%	52	65.82%		
Black/African-American					0.2	0.66
No	3330	93.07%	72	91.14%		
Yes	248	6.93%	7	8.86%		
Asian					8.88	0.003
No	3460	96.70%	71	89.87%		
Yes	118	3.30%	8	10.13%		
Hispanic					<0.001	1.00
No	3284	91.78%	73	92.41%		
Yes	294	8.22%	6	7.59%		
Other						0.45^
No	3378	94.41%	73	92.41%		
Yes	200	5.59%	6	7.59%		
Grade					1.74	0.63
9th	1091	30.49%	21	26.58%		
10th	972	27.17%	23	29.11%		
11th	955	26.69%	19	24.05%		
12th	560	15.65%	16	20.25%		
Family Structure						0.53^
One parent	825	23.06%	14	17.72%		
Two parents	2580	72.11%	61	77.22%		
Other	173	4.84%	4	5.06%		
Problematic Shopping						0.003^
No Problematic Shopping	3497	97.74%	72	91.14%		
Problematic Shopping	81	2.26%	7	8.86%		

PVG, problematic video gaming.

*p-values were calculated using chi-squared tests with Yates’ continuity correction, unless otherwise specified

^ p-values were calculated using Fisher’s Exact Test

**Table 3:** Sociodemographic characteristics of adolescents with problematic video gaming stratified by problematic-shopping status.

	Non-Problematic Shopping (N = 72)		Problematic Shopping (N = 7)		
Dependent Variable	N	%	N	%	p-value*
Gender					0.02
Male	62	86.11%	3	42.86%	
Female	10	13.89%	4	57.14%	
Race/Ethnicity					
White/Caucasian					0.41
No	26	36.11%	1	14.29%	
Yes	46	63.89%	6	85.71%	
Black/African-American					1.00
No	65	90.28%	7	100.00%	
Yes	7	9.72%	0	0.00%	
Asian					0.54
No	65	90.28%	6	85.71%	
Yes	7	9.72%	1	14.29%	
Hispanic					1.00
No	66	91.67%	7	100.00%	
Yes	6	8.33%	0	0.00%	
Other					1.00
No	66	91.67%	7	100.00%	
Yes	6	8.33%	0	0.00%	
Grade					0.39
9th	20	27.78%	1	14.29%	
10th	22	30.56%	1	14.29%	
11th	17	23.61%	2	28.57%	
12th	13	18.06%	3	42.86%	
Family Structure					0.32
One parent	14	19.44%	0	0.00%	
Two parents	54	75.00%	7	100.00%	
Other	4	5.56%	0	0.00%	

*p-values were calculated using Fisher’s Exact Test

STRAT was present in 9.6% of the sample (n = 350). S1 Table displays the sociodemographic characteristics of those with and without STRAT. Adolescents in the STRAT group were more likely to be female than those in the non-STRAT group. S3 Table shows the sociodemographic characteristics of adolescents with and without GTRAT. Adolescents in the GTRAT group, versus those in the non-GTRAT group, were more likely to be male and of Asian race/ethnicity, and were less likely to be Caucasian/White.

### PVG and PS

Video gaming characteristics in adolescents with and without PS are displayed in Table 4. Adolescents with versus those without PS were more likely to meet criteria for PVG (OR = 8.79, 95%CI [3.53, 21.86]). Adolescents in the PS group, compared to those not in the PS group, were also more likely to endorse attempts to cut back on video gaming (OR = 2.87, 95%CI [1.41, 5.85]), irresistible urges for video gaming (OR = 2.15, 95%CI [1.00, 4.64]), tension or anxiety relieved only by video gaming (OR = 3.67, 95%CI [1.76, 7.64]), a self-perceived problem with video gaming (OR = 4.27, 95%CI [1.79, 10.17]), family concern over their video gaming (OR = 2.12, 95%CI [1.02. 4.44]), and missing school, work, or activities to play video games (OR = 4.00, 95%CI [1.90, 8.41).

**Table 4:** Adjusted multivariate analysis of problematic video gaming in adolescents stratified by problematic-shopping status.

	Problematic Shopping vs. Non-Problematic Shopping		
Dependent Variable	OR	95%CI	p-value
Problematic Video Gaming	8.79	3.53, 21.86	<0.001
*Video Gaming Characteristics*			
Attempt to Reduce	2.87	1.41, 5.85	0.004
Perceived Problem	4.27	1.79, 10.17	0.001
Family Concern	2.12	1.02, 4.44	0.045
Missed School, Work, Activity	4.00	1.90, 8.41	<0.001
Irresistible Urges for Behavior	2.15	1.00, 4.64	0.05
Anxiety or Tension Relieved Only by Behavior	3.67	1.76, 7.64	<0.001

Multivariate analysis adjusted for sex, ethnicity, grade level, and family structure.

Relationships between each shopping measure and PVG status are displayed in Table 5. Adolescents who endorsed attempts to cut back on shopping (OR = 2.41, 95%CI [1.21, 4.80]), family concern over their shopping (OR = 2.37, 95%CI [1.18, 4.77]), missing school, work, or activities to shop (OR = 3.24, 95%CI [1.50, 7.01), a self-perceived problem with shopping (OR = 4.48, 95%CI [1.94, 10.33]), irresistible urges for shopping (OR = 3.59, 95%CI [1.76, 7.30]), and tension or anxiety relieved only by shopping (OR = 4.24, 95%CI [2.00, 8.99]) were more likely to meet criteria for PVG, relative to adolescents who denied those shopping measures.

**Table 5:** Adjusted multivariate analysis of problematic video gaming in adolescents stratified by each problematic-shopping measure.

	Problematic Video Gaming vs. Non-problematic Video Gaming		
Dependent Variable	OR	95%CI	p-value
*Shopping Measures*			
Attempt to Reduce	2.41	1.21, 4.80	0.01
Family Concern	2.37	1.18, 4.77	0.02
Missed School, Work, Activity	3.24	1.50, 7.01	0.003
Perceived Problem	4.48	1.94, 10.33	<0.001
Irresistible Urges for Behavior	3.59	1.76, 7.30	<0.001
Tension Relieved Only by Behavior	4.24	2.00, 8.99	<0.001

Multivariate analysis adjusted for sex, ethnicity, grade level, and family structure.

Results from exploratory analyses of the relationships between STRAT and PVG measures are displayed in S2 Table. Adolescents in the STRAT group were more likely to meet criteria for PVG than adolescents in the non-STRAT group (OR = 4.24, 95%CI [1.96, 8.88]). Adolescents in the STRAT group were also more likely to endorse irresistible urges for video gaming (OR = 2.36, 95%CI [1.48, 3.75]), tension or anxiety relieved only by video gaming (OR = 3.61, 95%CI [2.24, 5.83]), a self-perceived problem with video gaming (OR = 2.27, 95%CI [1.18, 4.37]), and missing school, work, or activities to play video games (OR = 3.28, 95%CI [1.97, 5.44). S4 Table displays results from exploratory analyses of the relationships between GTRAT and shopping measures. Adolescents in the GTRAT group were more likely to meet criteria for PS (OR = 3.64, 95%CI [1.85, 7.17]), family concern over shopping (OR = 2.49, 95%CI [1.65, 3.77]), missing school, work, or activities to shop (OR = 2.71, 95%CI [1.62, 4.52), endorse irresistible urges for shopping (OR = 2.49, 95%CI [1.48, 3.75]), and tension or anxiety relieved only by shopping (OR = 3.44, 95%CI [2.29, 5.18]).

### PVG health correlates and PS

Results from exploratory, interaction analyses assessing the effect of PS on relationships between PVG and health measures are shown in Table 6. The relationships between PVG and health correlates in adolescents with and without PS are displayed. Interaction ORs of PVG and PS showed that PS strengthened relationships between PVG and fighting (Interaction OR = 11.98, 95%CI [1.26, 114.13]), serious fighting resulting in physical injury (Interaction OR = 7.82, 95%CI [1.15, 53.27]), and weapon-carrying (Interaction OR = 40.76, 95%CI [3.96, 419.97.13]). Relationships between PVG and weapon carrying, as well as PVG and serious fighting resulting in injury, reached statistical significance in both PS and non-PS groups, while relationships between PVG and fighting did not.

**Table 6:** Adjusted Multivariate analyses of PVG on health/functioning measures stratified by problematic-shopping status.

	Non-Problematic Shopping			Problematic Shopping			Problematic shopping (PVG vs. Non-PVG) vs. Non Problematic Shopping (PVG vs. non-PVG) interaction		
	PVG vs. non-PVG			PVG vs. non-PVG					
Dependent Variable	OR	95%CI	p-value	OR	95%CI	p-value	Interaction OR	95%CI	p-value
Extracurricular Activities	0.84	0.50, 1.43	0.53	2.18	0.18, 25.81	0.54	0.91	0.15, 5.57	0.92
Lifetime Marijuana	0.95	0.56, 1.61	0.85	1.22	0.16, 9.18	0.85	1.75	0.27, 11.31	0.55
Lifetime Alcohol Binge	1.20	0.70, 2.04	0.5	0.37	0.06. 2.39	0.29	0.48	0.09, 2.63	0.4
Self-reported Alcohol Problem	1.95	0.75, 5.05	0.17	3.81	0.42, 34.90	0.24	5.53	0.80, 38.22	0.08
Regular Smoking	1.38	0.83, 2.29	0.22	0.84	0.07, 9.87	0.89	2.05	0.21, 20.27	0.54
Lifetime Drug Use	1.81	0.85, 3.84	0.12	3.91	0.20, 76.93	0.37	4.11	0.51, 32.80	0.18
Dysphoria	3.29	1.95, 5.55	<0.001	26.26	1.90, 363.08	0.01	4.35	0.46, 41.43	0.2
Fight	1.11	0.67, 1.83	0.69	8.85	0.78, 100.61	0.08	11.98	1.26, 114.13	0.03
Serious Fight	2.29	1.15, 4.55	0.02	13.09	1.04, 164.04	0.046	7.82	1.15, 53.27	0.04
Weapon Carrying	1.16	0.66. 2.02	0.61	72.89	4.88, 1089.80	0.002	40.76	3.96, 419.97	0.002

Multivariate analysis adjusted for sex, ethnicity, grade level, and family structure.

PVG, problematic video gaming.

## Discussion

PS and PVG are both behavioral addictions associated with significant impairment [3, 14]. This study is the first to demonstrate the association of PS and PVG in adolescents. This study is also the first to assess whether the co-occurrence of PVG and PS is associated with more negative health measures than in adolescents with PVG alone. PS was associated with increased odds of PVG and individual measures of PVG. Additionally, the presence of PS was associated with stronger relationships between PVG and aggression-related behaviors like getting into fights, serious fights resulting in injury, and weapon-carrying. Some implications of these findings are discussed below.

### PS and PVG

A striking finding of this study is that PS in adolescents was associated with significantly increased odds (OR = 8.79) of PVG. This suggests that the two behaviors are strongly related and video gaming behaviors should be assessed in adolescents with PS, and vice-versa. The mechanisms linking PS and PVG are still unexplored, but several possible links are considered below.

PS and PVG may co-occur in some adolescents due to underlying vulnerabilities for addictive behaviors. In our sample, PS was independently associated with addictive features of PVG, including seemingly uncontrollable urges for gaming, rising tension and anxiety just before gaming, unsuccessful attempts to cut back on gaming, and missing other activities to game. This may suggest that PS and PVG are linked by underlying neurobiological or psychological proclivities for addictive behaviors.

It is posited that negative reinforcement may contribute importantly to addictive behaviors, including PS and PVG, particularly for females [26], racial/ethnic minority groups [2], and in later stages of addictions [11]. In our sample, while endorsing any of the PS measures was independently associated with PVG, endorsing STRAT was one of the measures most strongly related to PVG, with a 4.24-fold elevated odds of PVG. Further analyses showed that STRAT was associated with higher odds of GTRAT, impairment due to video gaming, and irresistible urges for video gaming. Additionally, while GTRAT was associated with several shopping measures, it appeared most strongly related to STRAT. These data suggest that the relationship between PS and PVG may be driven by underlying negative reinforcement, as certain individuals engage in multiple addictive behaviors to relieve anxiety or tension. As such, targeting factors underlying negative reinforcement may help prevent co-occurring PS and PVG in adolescents. These efforts may be particularly important for females, given the higher frequency of STRAT among girls in the present study.

While there were only seven adolescents with both PS and PVG in our study, limiting some conclusions about the co-occurrence of the behaviors, those with PS and PVG were more likely to be female than those with PVG alone. The female predominance of this subgroup may also be due to proclivities towards negative reinforcement. Previous studies have demonstrated that specific groups including females (as well as individuals who have experienced trauma or marginalization and those with stress-related or affective disorders) may be more likely to experience negative reinforcement motivations to engage in addictive behaviors [26, 30]. Additionally, a previous study showed that gaming frequency is associated with increased anxiety symptomatology in females and decreased anxiety in males [31]. Taken together, these findings suggest that adolescents with concurrent PS and PVG may represent a distinct group than those with PVG alone, characterized by engagement in both behaviors to relieve anxiety, and therefore more likely to contain a higher percentage of females. These individuals may engage in PS and PVG to relieve anxiety and tension due to poor adaptive strategies for coping with emotional stress. Previous studies have shown that PS and self-injurious behaviors are linked in adolescents by their capacities for mood modification and relief from anxiety [18]. Similarly, PVG and PS have mood elevating qualities and may co-occur as maladaptive coping mechanisms in individuals with emotional dysregulation [13, 16]. Therefore, strategies to treat co-occurring PS and PVG may focus on cultivating alternative, more healthy coping strategies in adolescents.

Lastly, decreased inhibitory control has been implicated in behavioral addictions [11]. Both PS and PVG have been linked to impulsivity, which suggests that difficulties with impulse control may contribute to the development of both disorders [13, 14, 32]. In this sample, PS and PVG have both been linked to elevated impulsivity and sensation-seeking [7, 18]. Additional studies should examine more fully the relationships of impulsivity and sensation-seeking with negative-reinforcement motivations.

### PS, PVG, and health correlates

In this study, adolescents acknowledging PS demonstrated stronger relationships between PVG and aggression-related behaviors. These findings highlight the importance of identifying adolescents with PS among those with PVG. The mechanisms underlying the relationships between PS, PVG, and violence were not investigated in this study. However, as mentioned above, impulsivity and poor impulse control have been related to these behaviors, and PS may strengthen PVG’s relationship with violence, perhaps due to poorer impulse control in some adolescents [11, 33–35]. Particularly striking was the relationship between PVG and weapon-carrying among youth with PS (OR = 72.89) versus those without PS (OR = 1.16). Sensation-seeking has been associated with PS and has moderated the relationship between PVG and weapon-carrying in this sample [7, 18]. Sensation-seeking tendencies may underlie the relationship between PS, PVG, and aggressive/violent behaviors, and may be a suitable target for interventions. Further studies, especially longitudinal ones, are needed to investigate mechanisms underlying these relationships, and this information may help guide the development of more effective interventions.

### Strengths and limitations

Some of the strengths of this study include the use of a large sample size (n = 3,657), the use of multiple shopping and video gaming measures, and the measuring of associated health correlates for PS and PVG. However, the following limitations should be considered. First, certain limitations exist in the measures used. The survey data were collected in 2006, and since then, significant changes in video gaming and shopping have occurred. With the prominence of Amazon and online shopping, shopping behavior has shifted more from in-store to online, making these data less applicable for understanding current PS. At the time of survey distribution, online shopping was less popular and accessible than it is currently, and considerations of subject burden led the study team to exclude questions about online shopping. Additionally, changes in online gaming, including the increasing prominence of in-game purchasing, loot boxes/crates, and microtransactions have occurred. These changes in shopping and gaming may have strengthened or weakened the relationships between PS and PVG, or associated correlates. Future studies should incorporate measures for online shopping, particularly when studying the relationship between shopping and online gaming. However, the data provide a historic account against which more current findings may be compared. Next, although the study assessed the relationship between gaming and aggressive behaviors, the type of games played (such as violent games) was not assessed. Additionally, while the MIDI has been in the past to assess PVG and PS, more recently validated instruments were not included. In the current study, the MIDI had moderate reliability (with Kuder-Richardson ratios of 0.59 and 0.63 in PS and PVG, respectively). However, more recent tools have had better reliability and validity and should be considered in future studies [36–38]. Subject burden considerations at times led to decisions to use briefer assessments of some health and functioning measures in place of more detailed ones that may have had stronger psychometric properties. Nonetheless, questions from widely used assessments in youth (such as the Youth Risk Behaviors Survey) were employed to promote comparability across studies.

The data obtained also have certain limitations. While the overall sample size was large, the numbers of adolescents with PS and PVG were relatively low (88 and 79, respectively). As a result, some of the analyses may have been underpowered, and type II errors are possible. Additionally, the cross-sectional nature of the data and the absence of strong theoretical constructs to support a causal relationship between PS and PVG, prevented us from drawing causal inferences from the data. Future studies, preferably using longitudinal data, should study directionalities between PS, PVG, and their associated correlates. Lastly, while relationships between several behaviors have been demonstrated, the mechanisms underlying them have not been fully explored. As suggested by the I-PACE model and previous studies, positive reinforcement and mood elevation have been shown to contribute to both PVG and PS and may underlie the relationship between the two conditions [11, 14, 15]. However, the current study only investigated the role of negative reinforcement underlying the relationship between PS and PVG. Further studies should assess the role of other mechanisms underlying the relationships between PS, PVG, and aggressive/violent behaviors.

## Conclusions

Overall, the present study suggests that PS and PVG are strongly related in adolescents. Therefore, adolescents with one condition should be screened for the other. PS and PVG were also related by tendencies to engage in shopping or video gaming to relieve anxiety, suggesting that PS and PVG may be linked by motivations towards negative reinforcement, or underlying propensities for addictive behaviors. Lastly, comorbid PS strengthens the association of PVG and aggressive behaviors. Future studies should investigate features underlying PS, PVG, and aggressive behaviors for the development of interventions targeting these behaviors.

## Supporting information

S1 Table Chi-square analysis of sociodemographic characteristics of adolescents stratified by shopping-to-relieve-anxiety-or-tension (STRAT) status. (DOCX)

S2 Table Adjusted multivariate analysis of problematic video gaming in adolescents stratified by shopping-to-relieve-anxiety-or-tension status. (DOCX)

S3 Table Chi-square analysis of sociodemographic characteristics of adolescents stratified by gaming-to-relieve-anxiety-or-tension (GTRAT) status. (DOCX)

S4 Table Adjusted multivariate analysis of problematic shopping measures in adolescents stratified by gaming-to-relieve-anxiety-or-tension status. (DOCX)

S1 Text (TXT)

S1 Dataset (CSV)
