# Supplementary material for: An exploratory study of problematic shopping and problematic video gaming in adolescents
Source: PLoS One. 2022 Aug 10;17(8):e0272228. doi: 10.1371/journal.pone.0272228 (PMC9365157; doi:10.1371/journal.pone.0272228)
Supplement: S1 Table — (DOCX) [file pone.0272228.s001.docx]

Table S1

*Chi-square analysis of sociodemographic characteristics of adolescents stratified by shopping-to-relieve-anxiety-or-tension (STRAT) status*

|  | Non-STRAT (N= 3307) | | STRAT (N= 350) | |  |  |
| --- | --- | --- | --- | --- | --- | --- |
| Dependent Variable | N | % | N | % | χ2 | p |
| Gender |  |  |  |  | **114.63** | **<0.001** |
| Male | 1581 | 47.81% | 62 | 17.71% |  |  |
| Female | 1726 | 52.19% | 288 | 82.29% |  |  |
| Race/Ethnicity |  |  |  |  |  |  |
| White/Caucasian |  |  |  |  | 3.18 | 0.07 |
| No | 788 | 23.83% | 99 | 28.29% |  |  |
| Yes | 2519 | 76.17% | 251 | 71.71% |  |  |
| Black/African-American |  |  |  |  | <0.001 | 0.98 |
| No | 3077 | 93.05% | 325 | 92.86% |  |  |
| Yes | 230 | 6.95% | 25 | 7.14% |  |  |
| Asian |  |  |  |  | 0.36 | 0.55 |
| No | 3195 | 96.61% | 336 | 96.00% |  |  |
| Yes | 112 | 3.39% | 14 | 4.00% |  |  |
| Hispanic |  |  |  |  | 0.6 | 0.44 |
| No | 3040 | 91.93% | 317 | 90.57% |  |  |
| Yes | 267 | 8.07% | 33 | 9.43% |  |  |
| Other |  |  |  |  | 2.74 | 0.10 |
| No | 3128 | 94.59% | 323 | 92.29% |  |  |
| Yes | 179 | 5.41% | 27 | 7.71% |  |  |
| Grade |  |  |  |  | 0.29 | 0.96 |
| 9th | 1002 | 30.30% | 110 | 31.43% |  |  |
| 10th | 903 | 27.31% | 92 | 26.29% |  |  |
| 11th | 882 | 26.67% | 92 | 26.29% |  |  |
| 12th | 520 | 15.72% | 56 | 16.00% |  |  |
| Family Structure |  |  |  |  | 5.8 | 0.055 |
| One parent | 745 | 22.53% | 94 | 26.86% |  |  |
| Two parents | 2407 | 72.79% | 234 | 66.86% |  |  |
| Other | 155 | 4.69% | 22 | 6.29% |  |  |
| Problematic Video Gaming |  |  |  |  | 3.65 | 0.056 |
| Non-Problematic Video Gaming | 3241 | 98.00% | 337 | 96.29% |  |  |
| Problematic Video Gaming | 66 | 2.00% | 13 | 3.71% |  |  |
