# Supplementary material for: An exploratory study of problematic shopping and problematic video gaming in adolescents
Source: PLoS One. 2022 Aug 10;17(8):e0272228. doi: 10.1371/journal.pone.0272228 (PMC9365157; doi:10.1371/journal.pone.0272228)
Supplement: S2 Table — (DOCX) [file pone.0272228.s002.docx]

Table S2

*Adjusted multivariate analysis of problematic video gaming in adolescents stratified by shopping-to-relieve-anxiety-or-tension status*

|  |  | | STRAT vs. Non-STRAT | |  | |
| --- | --- | --- | --- | --- | --- | --- |
| Dependent Variable | OR | 95%CI | | p | |  |
| Problematic Video Gaming | 4.24 | 1.96 - 8.88 | | <0.001 | |  |
|  |  |  | |  | |  |
| *Video Gaming Characteristics* |  |  | |  | |  |
| Attempt to Reduce | 1.55 | 0.95 - 2.55 | | 0.08 | |  |
| Perceived Problem | 2.27 | 1.18 - 4.37 | | 0.014 | |  |
| Family Concern | 1.02 | 0.61 - 1.71 | | 0.93 | |  |
| Missed School,  Work, Activity | 3.28 | 1.97 - 5.44 | | <0.001 | |  |
| Irresistible Urges for Behavior | 2.36 | 1.48 - 3.75 | | <0.001 | |  |
| Anxiety or Tension Relieved Only by Behavior | 3.61 | 2.24 - 5.83 | | <0.001 | |  |
|  |  |  | |  | |  |
