# Supplementary material for: An exploratory study of problematic shopping and problematic video gaming in adolescents
Source: PLoS One. 2022 Aug 10;17(8):e0272228. doi: 10.1371/journal.pone.0272228 (PMC9365157; doi:10.1371/journal.pone.0272228)
Supplement: S3 Table — (DOCX) [file pone.0272228.s003.docx]

Table S3

*Chi-square analysis of sociodemographic characteristics of adolescents stratified by gaming-to-relieve-anxiety-or-tension (GTRAT) status*

|  | No Gaming to Relieve Anxiety or Tension (n = 3381 ) | | Gaming to Relieve Anxiety or Tension (n = 276 ) | |  |
| --- | --- | --- | --- | --- | --- |
| Dependent Variable | N | % | N | % | p* |
| Gender |  |  |  |  | **<0.001** |
| Male | 1418 | 41.94% | 225 | 81.52% |  |
| Female | 1963 | 58.06% | 51 | 18.48% |  |
| Race/Ethnicity |  |  |  |  |  |
| Caucasian |  |  |  |  | **<0.01** |
| No | 800 | 23.66% | 87 | 31.52% |  |
| Yes | 2581 | 76.34% | 189 | 68.48% |  |
| African-American |  |  |  |  | 1.00 |
| No | 3145 | 93.02% | 257 | 93.12% |  |
| Yes | 236 | 6.98% | 19 | 6.88% |  |
| Asian |  |  |  |  | **<0.001** |
| No | 3276 | 96.89% | 255 | 92.39% |  |
| Yes | 105 | 3.11% | 21 | 7.61% |  |
| Hispanic |  |  |  |  | 0.18 |
| No | 3110 | 91.98% | 247 | 89.49% |  |
| Yes | 271 | 8.02% | 29 | 10.51% |  |
| Other |  |  |  |  | 0.60 |
| No | 3193 | 94.44% | 258 | 93.48% |  |
| Yes | 188 | 5.56% | 18 | 6.52% |  |
| Grade |  |  |  |  | 0.44 |
| 9th | 1016 | 30.05% | 96 | 34.78% |  |
| 10th | 924 | 27.33% | 71 | 25.72% |  |
| 11th | 905 | 26.77% | 69 | 25.00% |  |
| 12th | 536 | 15.85% | 40 | 14.49% |  |
| Family Structure |  |  |  |  | 0.40 |
| One parent | 776 | 22.95% | 63 | 22.83% |  |
| Two parents | 2446 | 72.35% | 195 | 70.65% |  |
| Other | 159 | 4.70% | 18 | 6.52% |  |
|  |  |  |  |  |  |
