# Supplementary material for: An exploratory study of problematic shopping and problematic video gaming in adolescents
Source: PLoS One. 2022 Aug 10;17(8):e0272228. doi: 10.1371/journal.pone.0272228 (PMC9365157; doi:10.1371/journal.pone.0272228)
Supplement: S4 Table — (DOCX) [file pone.0272228.s004.docx]

Table S4

*Adjusted multivariate analysis of problematic shopping measures in adolescents stratified by gaming-to-relieve-anxiety-or-tension status*

|  | GTRAT vs. Non-GTRAT | | |
| --- | --- | --- | --- |
| Dependent Variable | OR | 95%CI | p |
| Problematic Shopping | 3.64 | 1.85 - 7.17 | <0.001 |
|  |  |  |  |
| *Shopping Characteristics* |  |  |  |
| Attempt to Reduce | 1.33 | 0.87 - 2.03 | 0.19 |
| Perceived Problem | 1.33 | 0.90 - 1.97 | 0.16 |
| Family Concern | 2.49 | 1.65 - 3.77 | <0.001 |
| Missed School, Work, Activity | 2.71 | 1.62 - 4.52 | <0.001 |
| Irresistible Urges for Behavior | 2.49 | 1.68 - 3.69 | <0.001 |
| Tension Relieved Only by Behavior | 3.44 | 2.29 - 5.18 | <0.001 |
